# Supplementary material for: EuRBPDB: a comprehensive resource for annotation, functional and oncological investigation of eukaryotic RNA binding proteins (RBPs)
Source: Nucleic Acids Res. 2019 Oct 10;48(D1):D307–13. doi: 10.1093/nar/gkz823 (PMC6943034; doi:10.1093/nar/gkz823)
Supplement: gkz823_Supplemental_Files [file gkz823_supplemental_files.zip › Supplementary Figure 1-3.pdf]

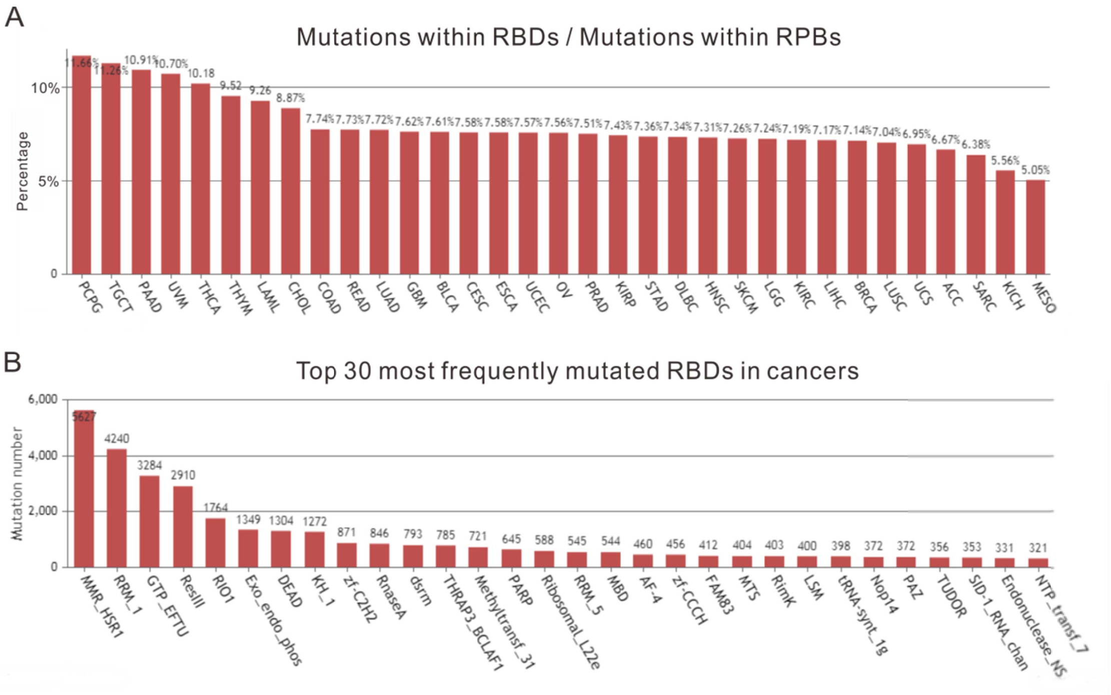

Supplementary Figure 1. (A) The percentage of the number of mutations within RBDs relative to total mutations within RBPs. **(B)** Top 30 most frequently mutated RBDs in 33 cancers.

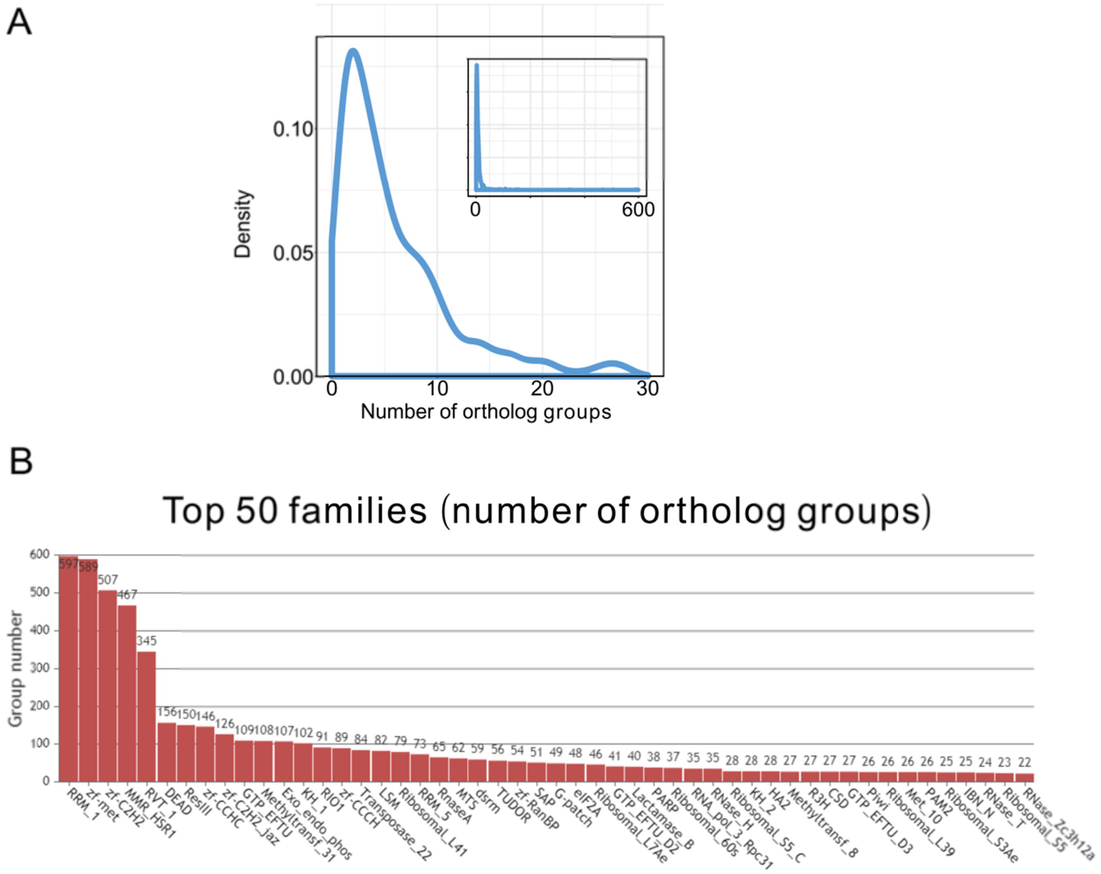

Supplementary Figure 2. (A) The distribution of the size of RBP families. **(B)** Top 50 families with highest number of ortholog group of RBP.

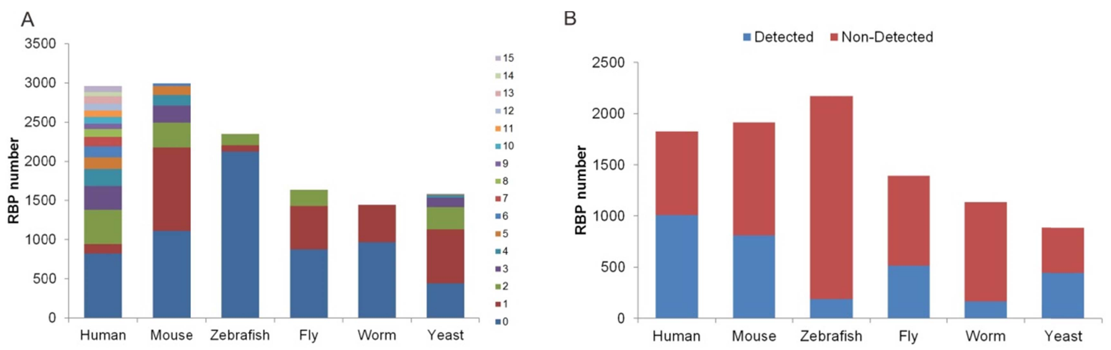

Supplemental Figure 3. The percentage of all RBP **(A)** and canonical RBP **(B)** detected by various RBPome methods.
